# CRISPR-Based Genome Editing: Advancements and Opportunities for Rice Improvement

**DOI:** 10.3390/ijms23084454

**Published:** 2022-04-18

**Authors:** Workie Anley Zegeye, Mesfin Tsegaw, Yingxin Zhang, Liyong Cao

**Affiliations:** 1 State Key Laboratory of Rice Biology, China National Rice Research Institute, Hangzhou 310006, China; workiean2009@gmail.com; 2 Department of Agricultural Biotechnology, Institute of Biotechnology, University of Gondar, Gondar P.O. Box 196, Ethiopia; mesfintsegaw@yahoo.com; 3 Institute of Crop Sciences, Chinese Academy of Agricultural Sciences, Beijing 100081, China; 4 Zhejiang Key Laboratory of Super Rice Research, Hangzhou 311400, China

**Keywords:** CRISPR/Cas9, Cas variants, genome editing, rice, improvements

## Abstract

To increase the potentiality of crop production for future food security, new technologies for plant breeding are required, including genome editing technology—being one of the most promising. Genome editing with the CRISPR/Cas system has attracted researchers in the last decade as a safer and easier tool for genome editing in a variety of living organisms including rice. Genome editing has transformed agriculture by reducing biotic and abiotic stresses and increasing yield. Recently, genome editing technologies have been developed quickly in order to avoid the challenges that genetically modified crops face. Developing transgenic-free edited plants without introducing foreign DNA has received regulatory approval in a number of countries. Several ongoing efforts from various countries are rapidly expanding to adopt the innovations. This review covers the mechanisms of CRISPR/Cas9, comparisons of CRISPR/Cas9 with other gene-editing technologies—including newly emerged Cas variants—and focuses on CRISPR/Cas9-targeted genes for rice crop improvement. We have further highlighted CRISPR/Cas9 vector construction model design and different bioinformatics tools for target site selection.

## 1. Introduction

Agriculture is currently facing many obstacles such as global climate change, a rapidly growing population, pests and pathogens, and many environmental threats. The growing population makes food security an urgent, pressing issue all over the world, and there it is forecast that global food-supply demand will double from 2005 to 2050 [1]. Rice is considered the main dietary component or primary source of food for a large portion of the world’s population. Several crops, including rice, have been domesticated and harvested for many years to meet the increasing demand for food. Nonetheless, attempts to guarantee food supplies by using traditional breeding methods have shown little promise. There is a critical need for more powerful technologies to improve foods’ nutritional content, increase abiotic stress tolerance, and enhance resistance to major biotic factors [2].

Recent advances in gene-editing technologies (GET) can contribute to coping with the complex problem of traditional breeding techniques, beginning a new age of crop enhancement. These methods allow us to alter genes of interest at specific locations and to provide new outlooks into crop functional genomics. A genome-editing system is a robust tool looking into the biological and biotechnological aspects of the characterization of plant genes and genetic modification. Recent genome-editing tools, such as zinc finger nucleases (ZFNs), transcription activator-like effector nucleases (TALENs), and clustered regularly interspaced short palindromic repeat (CRISPR)-associated endonuclease 9 (CRISPR/Cas9) techniques have opened up a new horizon for rice yield and quality enhancement [3,4,5]—of which, CRISPR/Cas9 has shown the most prominent potential for quick and effective genome editing in plant species. CRISPR/Cas9 genome editing as an advanced molecular biology tool enables us to carry out precise and efficient target modification, identifying novel prospects for evolving new plant variations using deletions, insertions, and substitutions, and hence encouraging the hastening of rice crop enhancement. So far, genome-editing tools employing CRISPR/Cas9 have been successfully exploited in rice for the improvement of drought tolerance [6], cold tolerance [7], salinity, and heavy metal stress [8,9].

More recently, newly emerged Cas variants such as CRISPR/Cpf1 (clustered regularly interspaced short palindromic repeat from Prevotella and Francisella 1) were developed in 2016. This technique is a more sophisticated and effective gene-editing technology than CRISPR/Cas9 [10]. As a new approach option that complements the HDR method, the strategy of a CRISPR/Cas9-based base editing enables the conversion of one base to another directly and permanently without a double-stranded break or donor DNA [11,12]. Researchers have successfully designed adenine base editors (ABE) and demonstrated the efficiency of A–T to G–C conversion using the ABE system in the rice genome [13]. Similarly, other scholars also confirmed the efficiency and precision of ABE by changing the A–T target into G–C without any other insertions/deletions (InDels) or substitution mutations in target sites, indicating its high efficiency in multiplex base editing [14]. Recent research has employed a fusion of CRISPR-Cas9 and activation-induced cytidine deaminase to develop multiple herbicide-resistant mutants [15,16,17].

Due to its small genome size, high transformation efficiency, accessibility of gene resources, and increased genomic synteny, rice is a model crop for functional and structural genomics studies. Thus, rice can be effectively exploited to examine multiple classes of rice genome modification in the CRISPR/Cas9 system [18,19], as well as to determine the function of functional genes [20,21,22]. To date, many studies have been released on the potential application of CRISPR/Cas9 in rice [23,24,25,26,27,28,29,30]. However, there is a need for an elaborative review, keeping in mind the rapid and extensive acceleration of new studies on the CRISPR/Cas9 genome-editing tool. Therefore, this article aims:

1. To discuss the current application of CRISPR/Cas9 to rice research that focuses on CRISPR/Cas9-targeted genes for rice crop improvement;
2. To discuss CRISPR/Cas9 components and mechanisms;
3. To highlight CRISPR/Cas9 vector construction model design and different online tools for target site design;
4. To compare CRISPR/Cas9 with other gene editing tools (ZFNs and TALENs), including the newly emerged Cas variants.

## 2. CRISPR/Cas9 System and Its Components

Initially, CRISPR/Cas9 was discovered as an adaptive immune system of archaea and bacteria. It was first reported by [31], and researchers discovered a means of exploiting it as a gene-editing technology. This technique can identify a particular site in a target gene in a highly efficient, unique, and flexible manner [32]. In roughly 50% of bacteria and 90% of archaea, CRISPR/Cas9 active immune mechanisms exist [33]. CRISPR/Cas9 is an endonuclease of DNA that splits the invading phage DNA into pieces and then incorporates it into the CRISPR set as a spacer. It was implemented efficiently in plants in 2013, and in five original research articles, the CRISPR/Cas9 scheme in rice was efficiently recorded [4,19,34,35,36]. Consequently, CRISPR/Cas9 technologies have proved to be an essential genome-editing method for rice.

**Figure 1:**
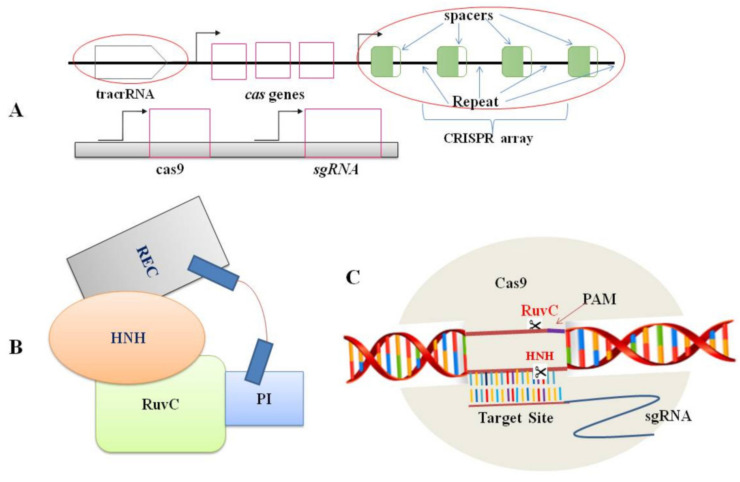
Components of CRISPR/Cas9 system: (**A**). Genomic structures of the CRISPR/Cas system (top) and the engineered CRISPR/Cas9 system (bottom); (**B**). A schematic representation of the Cas9 protein structure. Domains includes REC (large recognition lobe) and RuvC (a nuclease domain), which is linked with an arginine-rich region. HNH is a second nuclease domain. PI is PAM-interacting domain; (**C**). The conformation of the Cas9–sgRNA complex in the process of DNA cleavage. The Cas9 endonuclease is targeted to DNA by a guide RNA which can be supplied as a two-part system consisting of crRNA and tracrRNA or as a single guide RNA, where the crRNA and tracrRNA are connected by a linker. Target recognition is facilitated by the protospacer-adjacent motif (PAM). Cleavage occurs on both strands (scissors) 3 bp upstream of the PAM.

Nowadays, the editing system of CRISPR/Cas9 is the most common mechanism in plant biology for the genome editing process [37,38] because of its uncomplicatedness, flexibility, and efficiency. Two CRISPR/Cas9 components: the Cas9 protein and a short RNA molecule (sgRNA), together form a ribonucleoprotein complex (Figure 1). The sgRNA is made up of 18 to 21 nucleotides that are designed to target specific sites in the genome (protospacer). A G-rich (5′-NGG-3′) protospacer-adjacent motif (PAM) should be found downstream of the target site [31]. tracrRNA (trans-activating CRISPR RNA) and crRNA (CRISPR-derived RNA) are the two principal components of guide RNA. In essence, crRNA has homology area that enables it to integrate with tracrRNA. The tracrRNA has a stem-loop shape associated with the Cas9 protein. The crRNA and tracrRNA should be designed as sgRNA in the CRISPR/Cas9 gene-editing scheme to direct Cas9 dsDNA cleavage at the targeted site [31]. Following the appropriate site identification by sgRNA based on the Watson–Crick base-pairing rule, the Cas9-sgRNA complex moves through the genome and produces a double-stranded break (DSB) [33]. During cleavage site repairing, the error-prone non-homologous end-joining (NHEJ) pathway regularly leaves a lesion in the form of a minor InDel or substitution upstream of PAM. Such mutations can induce frame-shift mutations in the coding sequence of the gene and cause a premature stop codon, resulting in a loss or gain of function in mutants. This achievement has unlocked numerous other possibilities for scientists to obtain more knowledge on plant biological systems [39].

## 3. Mechanism of the CRISPR/Cas9 System

In three steps, namely adaptation/acquisition, expression, and interference, CRISPR/Cas9 identifies and targets the genetic material of foreign DNA [40,41,42,43]. The adaptation/acquisition phase includes the recognition, invasion, and binding of donor DNA that are cut into small segments and combined within the CRISPR locus. Then, the CRISPR locus is transcribed to create crRNA that directs the intended effect or endonucleases to attack viral items by complementary base pairing [44,45]. Since the protospacer contains a G-rich base pair (5′-NGG-3′), PAMs are used as recognition motifs for the adaptation/acquisition of the targeted site. During the second step of the CRISPR/Cas9 execution mechanism, the lengthy Pre-crRNA is deliberately transcribed from the CRISPR nucleus and reproduced into crRNAs using Cas9 proteins. Recently, researchers revealed that tracrRNA is also involved in Streptococcus pyogenes pre-crRNA processing [46]. The tracrRNA is associated with the repeat crRNA site through complementary base pairing and allows precrRNA to be processed in crRNA [47]. The processed crRNAs join the associated antiviral defense complex of CRISPR and help to identify and parse a particular target area of donor DNA [47]. At the final steps, i.e., interference, the system needs a Cas9 protein [48,49] so that the sgRNA can guide the cleavage of the Cas9 protein complex from the particular target area; this creates immunity from pathogen attacks [40,41].

## 4. The Advantages and Drawbacks of the CRISPR/Cas9 System

**Figure 2:**
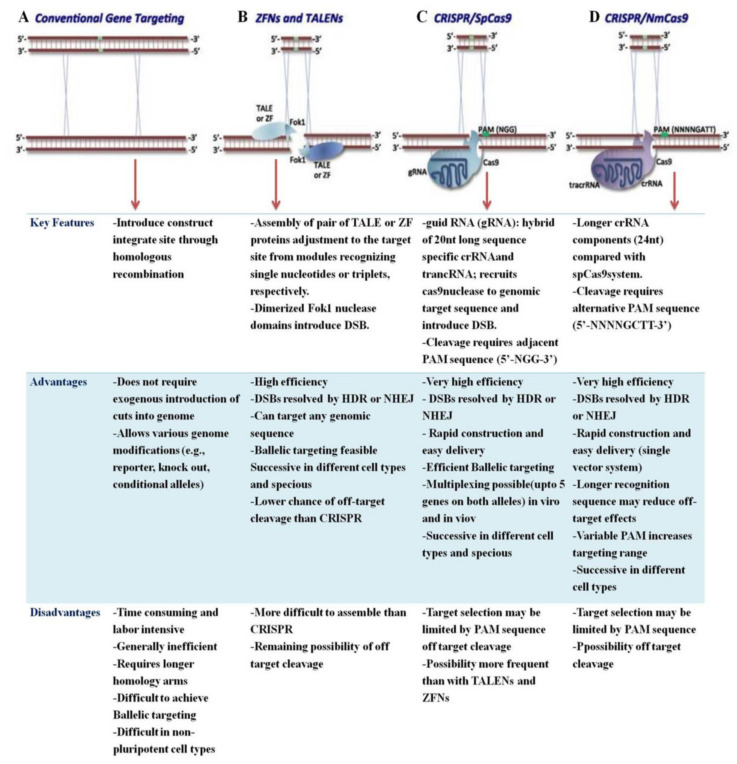
The advantages and disadvantages of the CRISPR/Cas9 system over other approaches for genome editing. (**A**). Conventional gene targeting. (**B**). ZNFs and TALENs. (**C**). CRISPR/SpCas9. (**D**). CRISPR/NmCas9. The red arrow indicates the corresponding gene editing method with its features, advantages, and disadvantages.

Until recently, the CRISPR/Cas9 tool was thought to be the best option for genome editing (GE) in plants, but it still has some drawbacks that limit its widespread application (Figure 2).

The following are a list of the major issues with CRISPR/Cas9 and the advantages of CRISPR/Cas variants:

1. The CRISPR/Cas9 system’s large size limits its editing efficiency, and it is not suitable for packing into viral vectors for delivery to somatic tissues. For efficient plant GE, a smaller-sized CRISPR/Cas is required;
2. SpCas9 involves a 5′-NGG-3′ PAM next to a 20 nt DNA target site where it only distinguishes the NGG PAM sequence, which limits its effectiveness when compared to new CRISPR/Cas variants. NG-Cas9 is more active and there is a newer variant, SpRYCas9 that is almost PAM-less. The broad PAM compatibility of SpRY greatly expands the targeting scope of CRISPR-based tools in plant genome engineering;
3. CRISPR/Cas9 has the potential to incorporate a large number of off-target mutations into the genome. However, by identifying various PAMs, new CRISPR/Cas variants have achieved better editing efficiency (fewer off-target mutations) of target bases in the sequence of interest;
4. CRISPR/Cas9 generates mutations at non-specific loci that are homologous to target sites;
5. CRISPR/Cas9-made mutant plants via Agrobacterium-mediated transformation systems are more expensive, time consuming, and resource intensive. The use of tissue culture-free genome editing systems, on the other hand, has the potential to improve efficiency;
6. The commercialization of transgenic crops expressing CRISPR/Cas9 faces challenges in a number of countries, owing primarily to development costs and constraints imposed by regulatory systems for the field release of genetically modified organisms.

## 5. Comparison of CRISPER/Cas9 Tools with ZFN and TALEN

Genome-editing methods are critical not only to explore gene functions in biology tasks but also to enhance the characteristics of genetic engineering in biotechnology. As shown in Table 1, we summarized the comparisons of CRISPER/Cas9 with ZFNs and TALENs.

ZFNs and TALENs are composed of DNA-binding protein and the enzyme FokI, whereas CRISPR binds together with Cas9 protein in the CRISPR/Cas9 system [23,50]. The DNA-binding domain of ZFNs or TALEs is comprised of a series of tandem repeat units; each one identifies and binds to one or more nucleotide targets on one strand of DNA. In the case of ZFN, each zinc finger repeat targets a triplet (3 nt), and yet internal and external context-dependent effects of adjacent fingers can influence the efficiency and specificity of the target domain relationships [18,51]. There is no accurate relationship, and several strategies have been developed to screen for and construct more efficient and specific ZFNs. TALE domains, on the other hand, exhibit a more predictable one-to-one correspondence between repeat units and their single-nucleotide targets, and no screening is required for TALEN engineering. Despite the fact that hundreds of ZFNs and TALENs have been constructed and tested, the targeting efficiency of these engineered endonucleases varies greatly. Indeed, the efficiency of TALENs in the same work or even targeting the same locus can range from low to high, or even show no targeting activity. Unfortunately, predicting the efficiency of a given ZFN of TALEN is still complicated, owing to a lack of sufficient knowledge about the properties of these engineered endonucleases. Through the comparison of all the evaluated TALENs or ZFNs, it may be possible to extract common rules regarding the efficiencies of these engineered endonucleases by analyzing the rapidly expanding information on these engineered endonucleases from a variety of sources. The specificity and potential toxicity of TALENs, on the other hand, require further careful exploration. ZFN technology is more mature in some applications, such as gene therapy trials, and it has recently been reported that ZFNs can be delivered directly into cultured cells in the form of purified proteins. Other factors in the engineering of customizable engineered endonucleases for TALENs and ZFNs, such as FokI cleavage domain variants and the relationship between the length of linker peptides and the length of spacers between the two engineered endonuclease monomer-binding sites can also influence the efficiency and specificity of these engineered endonucleases.

CRISPR/Cas9 gene-editing technologies continue to be an excellent tool in revolutionizing many areas of genome processing with their ease, effectiveness, and accuracy in the cleavage of target sites compared to TALEN and ZFNs [52,53]. In contrast to ZFN and TALEN, the specificity of the target DNA in CRISPR/Cas9 can be altered by programming the sgRNA sequence, and multiple sgRNAs can work with the same Cas9 protein for diverse targets at the same time [54]. Several papers have noted that effective changes have been carried out on several plant genes, but these techniques are comparatively tedious, costly, and only a few genes have been changed so far [55]. A CRISPR/Cas9 mechanism that was established using a Cas9 endonuclease and an RNA guide complex has demonstrated significantly higher effectiveness in gene editing. Additionally, CRISPR/Cas9 enables target identification by using gRNAs rather than DNA binding, which makes this method more reliable compared to ZFNs and TALEN [56]. While the specificity of CRISPR/Cas9 still requires further investigation, the frequency of off-target mutations is already less than that of physical and chemical mutagenesis methods [57].

## 6. Comparison of CRISPR/Cas9 with the Newly Emerging CRISPR/Cas GE Tools

**Figure 3:**
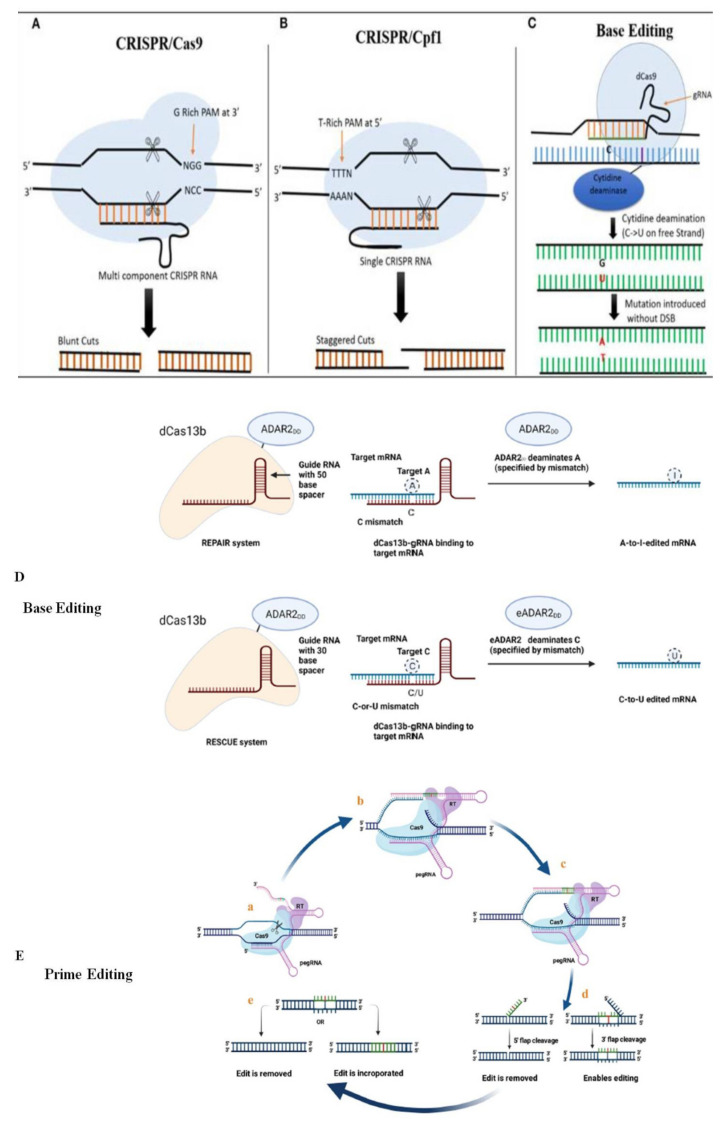
Comparison of CRISPR/Cas9 with newly emerging CRISPR/Cas GE tools. (**A**) In the CRISPR/Cas9 system, Cas9 is a multicomponent protein and recognizes a G-rich PAM at the 3′ end of the target site. Both tracrRNA and crRNA are required to recruit Cas9. Then, the Cas9 creates a DSB, resulting in blunt ends. (**B**) In the CRISPR/Cas12a System, Cas12a is a single-component protein which recognizes T-rich PAM at the 5′ end of the target sequence; tracrRNA is not required. The DSB results in a 5′ overhang sticky ends with staggered cuts. (**C**) In the nuclear base-editing system, cytidine deaminase fused with dCas9 is used to target the desired site. There is no DSB, cytidine deaminase converts C directly into U, and during mismatch repair a C→ T substitution can be corrected when the modified strand is used as template. (**D**) Base editing in RNA. In the REPAIR system, “A-to-I” editing uses dCas13 fused to ADAR2. REPAIR uses 50- nucleotide RNA with a 50-nucleotide mRNA–gRNA duplex. “A–C” mismatch in the RNA–gRNA duplex determines the target A. RESCUE system editing “C-to-U.” The optimum results are achieved with a gRNA with a 30-nucleotide spacer. The target “C” is specified by an induced “C–C” or “C–U” mismatch in the mRNA–gRNA duplex. (**E**) Prime editing. (a) Nicking the desired DNA sequence at the PAM strand by the fusion protein, (b) the exposed 3′ hydroxyl group primes the reverse transcription (RT) of the RT template of the prime editing gRNA (pegRNA), (c) reverse transcription, (d) the branched intermediate form containing two flaps of DNA: a 3′ flap (containing the edited sequence), and a 5′ flap (containing the dispensable, unedited DNA sequence) followed by flap cleavage, and (e) ligation and mismatch repair; either incorporating the edited strand or removing it.

Many recent studies have focused on improving the CRISPR/Cas9 system’s efficiency and accuracy by modifying it. New CRISPR/Cas tools (spCas9-NG, base editing, prime editing, xCas9, Cpf1, Cas13, and Cas14) are now being used for GE. These recently developed CRISPR/Cas variants with their potential applications are displayed in Figure 3 and Table 2.

The common Streptococcus pyogene Cas9 (SpCas9) recognizes canonical NGG PAM, limiting the rice genome’s editable range. Many studies have attempted to address this limitation by evaluating whether other Cas effectors (Cpf1 for AT-rich PAMs) and engineered Cas9 variants (VQR for NGA PAMs and VRER for NGCG PAMs) could be used in conjunction with other PAMs for rice genome editing [58]. Researchers have used stable transgenic lines to assess the efficacy of xCas9 and SpCas9-NG for gene editing in rice [59]. xCas9 was found to efficiently induce mutations at target sites in rice with NG and GAT PAM sequences.

Two types of xCas9 variants (xCas9 3.6 & xCas9 3.7) were created using PCR site-directed mutagenesis [60]. By creating 18 target sites containing GAA, GAT, and NG PAMs in three rice endogenous genes—MOC1, D14, and PDS—the feasibility of genome editing with xCas9 variants in rice was investigated [60]. The findings suggested that by utilizing some previously reported non-canonical PAMs, xCas9 could broaden the scope of genome editing in rice. The wild-type SpCas9 in the pCas9 (OsU6) vector was replaced with xCas9 3.6 and 3.7, yielding vectors pCas9 3.6 (OsU6) and pCas9 3.7 (OsU6). Overall, xCas9 3.7 outperformed xCas9 3.6 for genome editing in rice, demonstrating that xCas9 variants have the potential to become versatile tools that will broaden our understanding of genome editing.

SpCas9-NG, a SpCas9 variant rationally designed based on the structure of the SpCas9-sgRNA–DNA complex, has recently been found to recognize relaxed NG PAM sequences [61]. xCas9 and SpCas9-NG both recognize more relaxed PAM sequences when compared to SpCas9 [60,61]. xCas9 and SpCas9-NG variants that recognize relaxed NG PAMs can work more efficiently in rice, and SpCas9-NG is better suited for performing base editing in rice. The created versatile GE tools have significantly broadened the target scope in rice, benefiting basic plant research and crop genetic improvement [60]. xCas9, in general, has higher DNA specificity and editing efficiency, reduced off-target activity, and a greater PAM compatibility, unlike SpCas9 [60].

As demonstrated for rice [60] and multiple base mutations in rice [62], prime editing allows for the specific induction of insertions (up to 15 nt) and deletions (up to 40 nt). The pegRNA and fusion protein are introduced to the target cell, and once inside, the fusion protein nicks the cell’s DNA at the target sequence, initiating reverse transcription of the template sequence found in the pegRNA. The pegRNA and fusion protein are introduced into the target cell, and once inside, the fusion protein nicks the cell’s DNA at the target sequence, causing reverse transcription of the DNA template contained in the pegRNA to begin. As a result, an edited strand of DNA and an unedited strand of DNA are produced. The unedited strand is first removed, and then the newly edited strand is annealed back together to form double-stranded DNA.

Cas13 is a newly discovered CRISPR effector that can target specific viral RNAs and endogenous RNAs in plant cells [63]. Cas13 has a high level of RNA target specificity and efficiency [64]. Cas13 has been used to direct ADAR2 deaminase in human cells for RNA modification (changing adenosine to inosine) in order to recover functional proteins and halt disease progression [65]. CRISPR/Cas13a has recently been identified as an entirely new CRISPR type that belongs to class II type VI. It is attributed with RNase activity due to the existence of higher eukaryote and prokaryote nucleotide-binding (HEPN) domains [65].

Cas12a (Cpf1) and Cas12b (C2c1) from the class 2 type V CRISPR/Cas system, in addition to the Cas9 proteins, have been repurposed for GE [66]. They have many characteristics that distinguish them from Cas9 proteins; Cas12a and Cas12b recognize AT-rich PAM sequences, in contrast to Cas9, which recognizes GC-rich PAM sequences [66]. Cpf1 is dependent on a T-rich PAM sequence at the 5′-end of the protospacer sequence (5′-TTTN-3′ or 5′-TTTV-3′; V can be A, C, G). While Cas9 generates blunt-ended DNA breaks, Cpf1 generates DSBs with staggered ends at the distal position of a PAM, which may provide additional advantages—particularly for knock-in strategies—and may increase the effectiveness of NHEJ-based gene insertion [67].

Furthermore, Cpf1 is regarded as a better GE tool than CRISPR/Cas9 as it only demands a 42 nt crRNA, whereas Cas9 demands a 100 nt gRNA; meanwhile, Cpf1-mediated GE needs short sgRNA sequences [68]. Cpf1 proteins, which have RNase activity, have been used to process crRNA arrays for GE in plants [69]. These characteristics improve insertion efficiency at the Cpf1-cleaved site [70]. Recently, an improved Acidaminococcus sp. Cas12a variant (enAsCas12a) with a significantly expanded targeting range was engineered, allowing the targeting of previously inaccessible PAMs. When compared to sites with canonical TTTV PAMs, enAsCas12a exhibits a two-fold increase in GE activity. Cas14a, in contrast to other known class II systems, does not require a flanking sequence (PAM) near the target site [71]. In vitro validation of the PAM requirement demonstrated that Cas14a can cleave target sites regardless of the different sequences adjacent to the targets of these different guides [72].

Base editing (BE) is a remarkably new and diverse GE system that incorporates accurate and highly predictable nucleotide changes at genomic targets without the need for donor DNA templates, DSBs, or reliance on HDR and NHEJ [73]. It is thought to be more effective than HDR-mediated base-pair substitution because it results in fewer unwanted mutations at the target locus [74]. BE3 [75], BE4 [76], targeted-AID [70], and dCpf1-BE [77] are among the BE systems currently used to edit plant and animal genetic material. Such systems use Cas9 or Cpf1 variants to recruit cytidine deaminases, which use DNA mismatch repair pathways to generate specific C to T alterations. Furthermore, when directed by sgRNAs to genomic targets in human cells, the adenine base editors, which were created by fusing an evolved tRNA adenosine deaminase with SpCas9 nickase (D10A), convert A–T to G–C [78].

**Table 2:** Different newly emerged CRISPR/Cas techniques with potential functions in associated hosts.

Class Name	Size(AA)	PAM	Host	Spacer (bp)	Cut Site	Target	Ref
AacC2c1	1277	T-rich PAM	Alicyclobacillus acidoterrestris	20	Upstream of PAM	Ds DNA	[79]
CjCas9	984	NNNNACAC and NNNRYAC	Campylobacter jejuni	22	Upstream of PAM	DsDNA	[80]
Cpf1	–	TTTV	Prevotella & Francisella 1	20	Downstream of PAM	DsDNA	[67]
Cpf1(AsCpf1)	1307	5′-TTTN-3′	Acidaminococcus sp.	24	Downstream of PAM	DsDNA	[81]
Cas12a	-	Thymine-rich PAM sequences	Acidaminococcus sp.	-	Downstream of PAM	Ds DNA	[69]
Cas13	1440	Non-G nucleotide at the 3′	orthologs; Leptotrichia shaii	28	-	ssRNA	[82]
Cas14	400–700	-	Uncultivated archaea	-	-	ssDNA	[71]
FnCas9	1629	5′-NGG-3′	Francisella novicida	20	Upstream of PAM	Ds DNA	[83]
Nme Cas9	1082	5′ NNNNGATT-3′	Neisseria meningitidis	24 and 20	Upstream of PAM	DsDNA	[84]
SaCas9	1053	5ʹ-NNGRRT-3′	Staphylococcus aureus	21	Upstream of PAM	DsDNA	[85]
SpCas9	1368	5′-NGG-3′	Streptococcus pyogenes	-	Upstream of PAM	DsDNA	[31]
SpCas9-NG–	-	5′-NAC, NTG, NTT, and NCG	S. pyogenes	-	Upstream of PAM	DsDNA	[86]
St1Cas9	1121	NNAGAAW	Streptococcus thermophilus	20	Upstream of PAM	DsDNA	[87]
St3Cas9	1409	5′-NGGNG-3′	S. thermophilus	20	Upstream of PAM	DsDNA	[56]
xCas9	–	GAA, GAT and NG	-	19–22	Upstream of PAM	DsDNA	[88]

‘–’: information unavailable.

## 7. CRISPR/Cas9 Vector Design for Rice Genome Editing

CRISPR/Cas9 vector design and construction are comparatively easy and cheap. Components of the CRISPR/Cas9, namely crRNA and tracrRNA, together make up the sgRNA that directs Cas9 to implement target-specific DSBs. Effective CRISPR/Cas9 designing involves the transfer of both Cas9 and sgRNA proteins into target cells. They can be delivered via an agent (vectors) or directly (electroporation). The techniques of transmission in crops for gene editing using CRISPR/Cas9 approaches have been comprised of polyethylene glycol-mediated transformation, ballistics techniques, and Agrobacterium-mediated gene transformation [4,89,90]. For effective genome editing, it is essential to select ideal promoters to obtain the expression of sgRNA and Cas9 and codon-optimizedCas9. Codon-optimized SpCas9 variants in eukaryotic organisms have generally been used. Nevertheless, some studies have used the Cas9 option to optimize crop codons [4,35,89,90]. However, using RNPs or RNA, no optimization of elements is necessary for good expression (e.g., promoter, terminator, or codon optimization, etc.).

In many crops, the Cas9 expression is driven by by EF1A (Elongation factor 1-alpha), CMV (Cauliflower Mosaic Virus), or LTR (Long terminal repeats) promotors. Among these, 35S CMV (cauliflower mosaic virus) is the most common expression promoter [37]. The targeted gene for CRISPR/Cas9 needs a customized single guide RNA (sgRNA), which has a crRNA sequence and tracrRNA [31]. The crRNA structure gives the specificity of the targeted DNA, and it is feasible to design sgRNAs for multiplex gene editing with variable crRNAs. The crRNA region is a 20-nucleotide sequence homologous to the part of the gene that you are interested in, and that directs the activities of the Cas9 nuclease [91]. The target DNA sequence is, therefore, 20 bp followed by a PAM (NGG/NAG) sequence. However, DNA targets and crRNAs that show a discrepancy from the recognized 20 bp length have also been reported in a few studies, with length variations ranging from 19 to 22 bp [4,19,35,37]. Consequently, (N) 19–22 NGG or (N) 19–22 NAG is the target DNA sequence. Nevertheless, CRISPR/Cas9 may be restricted by the accessibility of the PAM site when targeting a specified sequence [92]. The rice genome demonstrates an abundance of potential PAM (1 in 10 bp) sites [35], which allows CRISPR technology to be used rapidly in the rice genome.

The U6 and U3 plant RNA III promoters have been used for sgRNA expression [37]. These promoters have to initiative nucleotide “G” and “A” transcriptions, respectively. Thus, the sgRNA crRNA structure for the promoter U6 is G (N) 19–22, and the promoter U3 is A (N) 19–22. The initiating G and A are often fused directly to the gRNA or are part of the target sequence [4,19,35,36,37,90].

**Figure 4:**
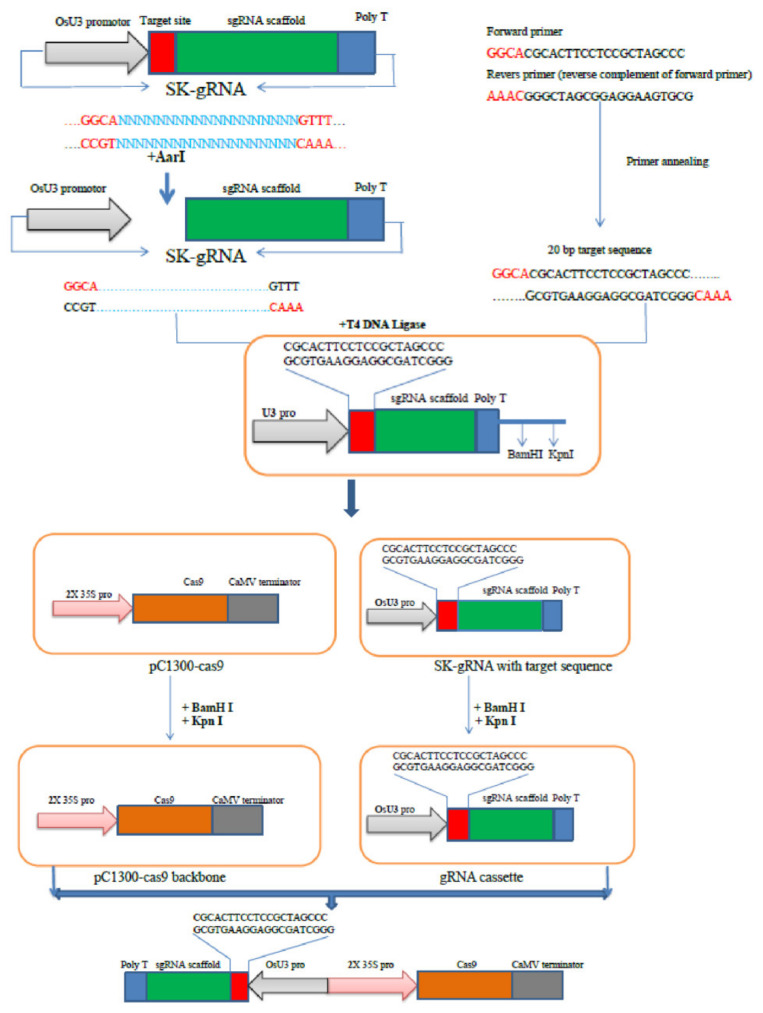
T-DNA vector with all the components necessary for Cas9-induced mutagenesis. The 20 bp protospacer sequences of each target site are subcloned or integrated between the sgRNA scaffold and the *U3* promoter by ligation of primers into an *AarI*-digested SK-sgRNA vector. Then, this vector is again re-ligated with a pC1300-Cas9 vector by ligation of *BamHI* and *KnpI*-digested enzymes. The whole sgRNA cassette is then delivered into a pC1300-Cas9 vector (contains the *Cas9* gene under the control of the 2 × 35S promoter) for plant transformation.

Here we outline a procedure for performing targeted editing employing the CRISPR/Cas9 system (Figure 4). The first procedure is the selection of a target site to be placed at the short PAM sequence at its 3′ end or downstream of its protospacer. The choice of the target site is an essential focus of large-scale CRISPR/Cas9 technology applications. The choice of the appropriate target site(s) can achieve the lowest/no off-target effects at an important mutation site. Many bioinformatics online tools for the designing of sgRNA as well as the identification of off-target effects are available in model organisms, including rice crops [93].

Following the identification of a target site, the next step is to design the target site-related oligonucleotide or primers. A DNA fragment encoding the sgRNA scaffold, which is placed under the appropriate promoter to optimize expression, is fused into the developed primers. For cloning, appropriate sequences of adaptors should be included. The inverse primary should have a 5′-AAAC-3′ adaptor, while the forward primer must have 5′-GGCA-3 adaptors (Figure 4). Once the protospacer sequence is subcloned into an SK-sgRNA vector or cassette (an example vector for our case study), the next step is inserting the constructs into the Cas9 expression vector (pC1300 Cas9), followed by its delivery into the rice plant cells by appropriate methods such as Agrobacterium transformation, protoplast transformation, or callus bombardment.

## 8. Important Considerations for gRNA Selection

The best techniques for gRNA selection—through experiment development, execution, and evaluation, are always essential to the correct use of CRISPR technology. The best sgRNA thus relies a great deal on what one is attempting to do when developing sgRNAs for use in the CRISPR technique: gene knockout, a editing of a particular base, or gene expression modulation.

The following variables should be regarded for the design of sgRNAs:

1. The DSB should be introduced near to the 5′ end of the coding region or in the indispensable domains. If choosing a target sequence in the proximity of the adjacent protospacer motif (PAM), the 3′ end of the target sequence must have a PAM sequence (5′-NGG-3′; Figure 4). The target sequence (crRNA) should be upstream of the PAM structure, and the Cas9 nuclease will digest around three bases upstream of the PAM. For cleavages, the PAM sequence is compulsory; however, it is not a component of the sgRNA sequence and should, therefore, not be included in the sgRNA sequence itself.
2. When selecting the right target sequence for on-target activity, targets that have poly-T and very low or very high GC content (≤25% or ≥80%) have low editing efficiency. Similarly, target site(s) with eight or more adjacent nucleotides have low editing effectiveness and should be coupled to the sgRNA sequence [94].
3. Another consideration before the start of the CRISPR experiment is decreasing off-target effects. It is not always so important in what the location the gene target is, but the gRNA sequence must be designed to be highly active and minimize off-target sites. Potential off-target sites with higher scores may have a higher probability of being targeted by the sgRNA/Cas9 nuclease complex. Thus, the selected gRNA spacers/target sequence should have sufficient specificity to avoid off-target editing.

In general, designing the CRISPR/Cas9 vector for any experiment needs to balance the maximization of on-target activity while minimizing off-target activity, which sounds obvious.

## 9. Bioinformatics Tools Available for sgRNA Designing

Many bioinformatics tools for sgRNA designing with high specificity and off-target detection in model organisms have been created (Table 3). After considering SNP and/or InDel in the genome, a unique platform for selecting sgRNA with significant effects is required [95]. More recently, highly specific sgRNAs with few or no off-target results were executed in rice, and a CRISPR–GE platform has been developed [94].

At present, the technique is being used as a powerful and multipurpose technology for genome-engineering, such as editing “changing the genomic sequence” [56,96] and regulation “repressing or activating expression of genes” [97]. For accurate and programmable gene targeting, a single guide RNA (sgRNA) is needed [75]. Practical and specific gene editing demands a detailed layout of sgRNAs, which is still a significant task. In this review, we listed some of the computational tools (Table 3) that have been used to assist in the design of sgRNAs for the CRISPR/Cas9 editing system.

**Table 3:** Available bioinformatics tools for sgRNA designing.

Tool Name	Link	Reference
CRISPR-GE	http://skl.scau.edu.cn/	[94]
CRISPRdirect	http://crispr.dbcls.jp/	[98]
CRISPR-P	http://cbi.hzau.edu.cn/crispr/	[99]
CRISPResso	http://crispresso.rocks/	[100]
E-CRISP	http://www.e-crisp.org/E-CRISP/	[101]
BreakingCas	http://bioinfogp.cnb.csic.es/tools/breakingcas/index.php	[102]
CRISPR-DO	http://cistrome.org/crispr/	[103]
CRISPOR	http://crispor.tefor.net/	[104]
CT-Finder	http://bioinfolab.miamioh.edu/ct-finder/	[105]
sgRNACas9	http://www.biootools.com/	[106]
CRISPR design	http://crispr.mit.edu/	[107]
Cas9 design	http://Cas9.cbi.pku.edu.cn/	[95,108]
Cas-Designer	http://www.rgenome.net/cas-designer/	[109]
CGAT	http://cbc.gdcb.iastate.edu/cgat/	[108]
Cas-OFFinder	http://www.rgenome.net/cas-offinder/	[110]
CCTop	http://crispr.cos.uni-heidelberg.de/	[111]
ProtospacerWB	http://www.protospacer.com/	[112]
SSC	http://crispr.dfci.harvard.edu/SSC/	[113]
CRISPR multi targeter	http://www.multicrispr.net/	[114]
MAGeCK	https://sourceforge.net/p/mageck/wiki/Home/	[115]
GT-Scan	http://gt-scan.braembl.org.au/gt-scan/	[116]
GuideScan	http://www.guidescan.com/	[101]
CrisprGE*	http://crdd.osdd.net/servers/crisprge/	[117]

‘–’: information unavailable.

## 10. Applications of CRISPR/Cas9 Gene-Editing Technologies in Rice

To keep pace with food consumer demand, the potential increase of rice yield and quality depends on the integration of multiple modern breeding approaches, comprehensive agronomic practices, and appropriate social and economic policies to simulate crop production activities. Therefore, the enhancement of rice yield and quality using revolutionary breeding methods is instantly desirable in order to raise access to nutritious foods at a global level for the ever-increasing population. Recently, many research works have shown the application of CRISPR/Cas9 genome editing strategies in rice, covering several aspects from biotic stress resistance to abiotic stress tolerance, and the achievement of improved yield performance, biofortification, and improvements in quality (Table 4). In this paper, the potential applications of CRISPR/Cas9 in rice genome editing are taken into consideration in attaining a large number of objectives.

### 10.1. Improving Rice Yield and Quality

Improving the quality of food is important as many individuals depend on rice as their basic sustenance. However, improvements via conventional methods have limitations in terms of being time consuming and challenging, causing off-target changes, the involvement of undesirable traits, and their lower efficiency. CRISPR/Cas9 is a useful tool for knocking out negative regulators controlling yield-associated characteristics of rice, such as grain weight (OsGW5, OsGLW2), grain size (OsGS3), grain numbers (OsGn1a), tiller number (OsAAP3), and panicle size (OsDEP) [120,123,145,146]. The simultaneous knocking out of three rice grain weight genes, namely TGW6, GW2, and GW5, resulted in a significant enhancement of grain weight characteristics [22]. Since most yield-related components are quantitative and influenced by the environment, the knockout of individual variables may not be enough to boost yield. Recently, it was discovered that editing the OsSPL16 gene with CRISPR/Cas9 improves grain yield by modulating the expression of pyruvate enzymes and cell cycle proteins [147].

Improving rice grain quality characteristics via current advanced genome-editing tools is a rapid, feasible, and economical approach [148]. Several rice genes that determine grain quality appearance have been isolated. Grain appearance and chalkiness are among the quality traits that impact the market adequacy of rice [149,150]. The integration of functionally characterized genes such as OsSPL13, OsSPL16/GW8, Chalk5, and GW7 using CRISPR/Cas9 and the assessment of their relationships with other genes can significantly enhance our understanding of rice grain appearance and milling quality.

The Waxy gene in the endosperm is primarily responsible for the amylose content (AC), which determines the cooking and eating quality of rice grains [151]. Waxy was knocked out using CRISPR/Cas9, resulting in lower amylose content and, as a result, improved rice quality for food and high cooking efficiency [138,152]. Several studies have also been conducted to determine the roles of various enzymes or genes involved in transgenic Taichung65 rice plants comprising a Waxy antisense construction, which had lower AC; transgenic plants were also demonstrated to have lower AC levels in hybrids [153]. The SBEIIb gene has also been knocked out via CRISPR/Cas9 to create high amylose rice, which is an ideal food for patients with diet-associated noninfectious diseases [139]. The successful editing of the BADH2 gene, responsible for fragrant aromatic rice, involved the insertion of one nucleotide in the mutated line, which showed a significant amount of 2AP and enriched the fragrance of the rice [127]. Protoplast and particle-bombarded rice calli systems were used to validate sequence-specific CRISPR/Cas9-mediated genomic alteration of three rice genes, OsPDS, OsBADH2, and OsMPK2, which controlled the responses to various abiotic stress stimuli [4]. Editing rates of approximately 7% and 9% for OsBADH2 and OsPDS were observed, respectively. Similarly, the five carotenoid catabolic genes, namely; OsCYP97A4, OsDSM2, OsCCD4a, OsCCD4b, and OsCCD7, have been successfully knocked out to improve the quantity of β-carotene in rice endosperm [154].

### 10.2. CRISPR/Cas9 Systems in Developing Climate Resiliance

Climate-resilient farming for contesting biotic and abiotic stress is an upcoming crop enhancement of interest using genome editing approaches [155,156]. The CRISPR/Cas9 RNA-led method is regularly used to improve plants, but it has been considered in very few papers so far in producing climate-resilient plants. The primary influences on crop yield and performance are stresses. Many crops, including fungal, bacterial, and viral resistance and insects with enhanced biotic stress resistance, were acquired by the knockout of CRISPR/Cas9. Scientists have moved from proof-of-concept to more applicative uses of CRISPR/Cas9 in rice. Multiple disease resistance lines have been obtained via this technology. Blast is a critical fungal disease in rice, but resistance genes against this disease have been developed by targeting the OsERF922 gene [20]; these findings revealed that there was a considerable decrease in blast lesion formation under pathogen infection. Similarly, knocking out of the Bsrk-1 gene enhanced the resistance of rice against blast without compromising yield [128]. The knocking-out of OsSWEET13 resulted in crops that resist rice bacterial blight caused by *Xanthomonas oryzae* pv. *oryzae* (*Xoo*) [129]. A recent study [157] has discovered that OsCYP71A1 disruptively blocks serotonin biosynthesis with considerably higher concentrations of salicylic acid, giving resistance to planthoppers and stem borers–two of the most rice-damaging pests.

Contamination of arable lands is one of the abiotic stresses that have generated the need to avoid the buildup of poisonous heavy metals in plants. By knocking out OsARM1, OsNramp5, and OsHAK1, researchers were able to identify rice species with high cadmium and arsenic concentrations [8,136,158]. In 2018, a study of the OsPYL abscisic acid receptor family showed that the triple knockout of ply 1/4/6 by CRISPR/Cas9 produced improved grain output, enhanced tolerance to high temperatures, and lower pre-harvest sprouts [124]. The suppression of the cadmium (Cd) OsNramp5 transporter gene resulted in the growth of low-Cd hybrid rice populations. CD accumulation in roots, seeds, and shoots was reduced by mutant osnramp5 [8].

Scientists have also been able to generate CRISPR/Cas9-mediated herbicide-resistant rice plants [133]. Studies into herbicide resistance were started to guarantee public and environmental health, as both are affected by agrochemical use [159]. ALS1 is one of the essential enzymes accountable for herbicide resistance in rice. Its function was disturbed using CRISPR/Cas9 at multiple discrete points [133], and the results showed that homology-directed repair mediated by CRISPR/Cas9 was effective. The second exon of BEL was knocked out by CRISPR/Cas9 in the rice cultivar Nipponbare, which was associated with bentazon and herbicide resistance [21]. CRISPR/Cas9 was used to edit TIFY1b [160], a transcription factor, and the OsAnn3 gene [7], which considerably improved cold tolerance. However, a great potential remains to be utilized; there is a considerable deal of plant genome manipulation that has been carried out through genome editing using the CRISPR/Cas9 system. Resistance to imazapic (IMP) and imazethapyr (IMT) was conferred in rice mutant plants by CRISPR/Cas9-mediated knockout of the Acetolactate Synthase (OsALS) gene [161]. In general, the CRISRR/Cas9 system has been successfully used to improve abiotic stress tolerance in rice [162].

### 10.3. Practical Approach for Hybrid Seed Production

Hybrid breeding or heterosis is a powerful approach used in increasing the productivity and yield of crops. Evolving a male sterile maternal line is a precondition required for developing a high-quality hybrid variety [163]. Hence, marvelous achievements have been achieved via CRISPR/Cas9-mediated gene knockout in the development of rice male sterile lines, including thermosensitive male sterile tms5 lines [121,164], photosensitive genic male sterile csa rice [123], and TMS10 [122]. Rice photosensitive and thermosensitive male sterile lines have been developed to accelerate and exploit heterosis breeding [121,165]. However, hybrid sterility is a crucial bottleneck in the exploitation of heterosis in rice breeding. To overwhelm the generative barriers, SaF/SaMatSa and OgTPR1 at the S1 locus [166] were disrupted in japonica–indica hybrids. The recovery of male fertility in japonica–indica hybrids was demonstrated through the knockout of one or two copies of the Sc gene in the indica allele Sc-I [167]. TMS10, created by genetic crosses or genome editing, is a temperature-sensitive male sterile mutant applicable to hybrid seed production [122]. The results provide insights into how rice copes with adverse changes in temperature to attain normal male fertility and a new breeding line for rice hybrid seed production. Correspondingly, knockout of the ORF2 toxin gene, which is accountable for the newly revealed selfish gene suicide mechanism of rice, rescued the fertility of japonica–Indica hybrids [168].

Another obstacle for exploiting hybrid breeding is that heterosis is lost in the following generations because of genetic segregation. Besides this, the high cost of hybrid seed production hinders the application of heterosis in many crops, including rice. Two independent groups [169,170] used a multiplex CRISPR/Cas9 editing system to replace meiosis by mitosis in rice through the knockout of three essential meiotic genes: REC8, PAIR1, and OSD1. They developed asexual propagation lines either by simultaneous activation of BBM1 in the egg cell or by knocking out MTL, respectively, enabling the repair of heterozygosity of hybrids through clonal seed propagation. The study results showed that the MiMe phenotype could be quickly introduced into hybrid rice varieties using CRISPR/Cas9 genome-editing technologies.

### 10.4. Rapid Generation of Genetic Diversity

Furthermore, CRISPR/Cas9 applications include comprehensive genetic diversity studies. Germplasm is a fundamental tool for the enhancement of crops in sustainable farming. The main requirement of the plant breeder is the generation of trait-specific genetically varied relatives for improving the characteristics of the crop. The main objective of breeders is to concentrate studies into the collection, preservation, and identification of new elite germplasms, which eventually produce agriculturally superior cultivars with a broad genetic foundation in breeding programs.

Genetic diversity is an essential cause of plant characteristic enhancement. The creation of genetic variables in the gene pool is the precondition for the development of distinctive plant crops. Transgenes in the enhanced variations can be removed once the necessary changes have been made. Traditional plant improvement methods rely heavily on genetic differences between natural germplasms. The introduction of positive characteristics into the chosen germplasm necessitates a chronological overview, as well as a time and energy-intensive evaluation of the vast population [148].

Although mutation breeding methods can be significantly accelerated and directed towards more than 2250 new plant variants [171], subsequent modifications in the mutated genes are random. This is why plant biologists have sought methods that can easily edit required genes of interest. Hence, reverse genetic methods increase the progress of crop improvement through targeted gene editing, and the CRISPR/Cas9 gene-editing system is an advancing approach for the rapid introduction of population diversity in rice crop breeding and development [47].

CRISPR/Cas9 now makes it simple to edit the multiplex genome of a possibly limitless number of genes [172] and has been recently verified in Arabidopsis and rice [173,174,175]. Eight rice genes such as BADH2, DEP1, Gn1a, GS3, GW 2, Hd1, EP3, and LPA1 have been successfully edited using one binary vector [174]. They provided a strategy trial for the rapid generation of various breeding resources, targeting these agronomically essential rice genes, using CRISPR/Cas9 multiplex genome-editing technology. DEP1, EP3, Gn1a, GS3, and GW2 are yield-associated genes; LPA1 is a plant architecture gene; while BADH2 and Hd1 are associated with rice fragrance and photoperiod genes, respectively. The knockout of genes that have an enormous effect on grain yields, such as GS3, DEP1, GS5, GW2, Gnla, and TGW6, is an easy and direct method for average rice output. The mutants of these genes produce the desired, convincing phenotypes [120,176]. The thousand-grain weight (TGW) increased significantly through the development of triple rice mutants by the simultaneous knocking out of GW2, GW5, and TGW6 [22]. Four genes were recognized as panicle architecture, plant architecture, seed size, and grain number regulators by a study team centered in Guangzhou (China), demonstrating that various agronomically suitable characteristics can be rapidly enhanced in a single cultivar [120].

CRISPR/Cas9 could also provide an efficient pyramid breeding technique with the simultaneous alteration of several characteristics [23,177]. Consequently, CRISPR/Cas9 is a potential tool for feeding the world’s population and achieving the goal of zero global hunger [178]. The insertion of big sequences via NHEJ would enable transgenes to be introduced into a specified locus, which supports high-level transcripts and does not interfere with endogenous gene function. Site-specific nucleases allow the addition of many genes near the current transgenic nucleus to achieve focused molecular feature stacking. It also allows the introduction of several characteristics in plants that have a small degree of segregation, which is hard to achieve through standard breeding or even genetic engineering [179]. There is also a tremendous potential for CRISPR screens to map and interrogate gene regulatory networks at an unprecedented speed and scale. The implementation of CRISPR screens offers new opportunities to analyze plant genomes at deeper resolution and will greatly advance plant functional and synthetic biology [180]. Researchers [181] have developed genomic resources and efficient transformations in the orphan Solanaceae crop ‘groundcherry’ (*Physalis pruinosa*) and have used CRISPR–Cas9 to mutate orthologues of tomato domestication and improvement genes that control plant architecture, flower production, and fruit size, thereby improving these major productivity traits. Accordingly, translating knowledge from model crops enables the rapid creation of targeted allelic diversity and novel breeding germplasms in distantly related orphan crops. More importantly, promoting integrative ecological (biodiversity resources) genomic studies promises a better understanding of antagonistic co-evolutionary interactions, as well as the more efficient breeding utilization of resistant phenotypes. In this case, gene prediction may also go beyond pre-breeding efforts and feedback on restoration optimization [182].

## 11. Conclusions Remarks

The progress of science is dependent on new techniques, discoveries, and ideas, so the development of novel tools and techniques is essential for scientific advancement. Plant breeding has been transformed by the advancement of genome-editing technologies. Continuous innovation in crop breeding and genetics is essential in meeting challenges and achieving sustainable food production. CRISPR, a cutting-edge molecular biology technique, has already broadened our understanding of genome regulation and organization in living cells from various biological kingdoms. CRISPR is revolutionizing not only agriculture, but also industry, the environment, medicine, and other fields. Due to the relative specificity of each nuclease platform, the majority of research work using the CRISPR/Cas9 genome-editing technique has been groundwork/preliminary to date. In the future, the use of high-throughput methods that allow for the comprehensive profiling of off-target cleavage sites should provide insight into the target recognition stringency inherent in each system. As a result, more advancement is required to fully exploit the platform, which will result in increased on-target efficiency. Many strategies, including modifying Cas9 to recognize different PAM sequences, have been used to overcome this limitation. The xCas9 3.7 variant was created to broaden the scope of genome editing in rice. A new version of Cas9 (SpCas9) was also created that could recognize NG PAMs in rice. Many other such variants are ineffective in plants, emphasizing the need to develop more Cas9 variants capable of recognizing a wide range of PAMs. The limitation imposed by PAM specificity can also be overcome by using the newly discovered Cas14a system, which does not require PAM, but can only target ssDNA. As a result, the rapid advancement of research into epigenetic genome modifications in rice is a promising approach for future rice crop improvement. As the scope and power of CRISPR technologies expand, social and ethical concerns about their use grow, and the applications of these powerful tools deserve more thought.

## Figures and Tables

**Table 1:** Comparison of CRISPER/Cas9 with ZFN and TALEN.

Editing Technology	DNA Binding Determinant	Endonuclease	Target Length (bp)	Off Targeting	Intended Effects	Unintended Effects
CRISPR/Cas9	crRNA/sgRNA	Cas9	18–21	Variable	Highly specific, highly efficient, and multiple targeting sites	Target selection limited by the requirement for PAM sequences; Off-target effects
ZFN	Zinc finger protein, FokI1018	FokI	18–36	High	Any genomic sequence targeted; Fewer off-target effects	Low efficiency; Targets only a single site at one time
TALENs	Transcription-activator like effector	FokI	30–40	Low	Targets any genomic sequence; Off-target effects are limited	Comparatively low inefficiency; Targets only a single site at one time; Sensitive to target DNA methylation

**Table 4:** A list of genes involved in a variety of agriculturally relevant parameters targeted by CRISPR/Cas9.

Application Perspectives	Targeted Genes	Molecular Functions	Cas9 Promoter	sgRNA Promoter	Transformation Method	References
Yield and quality improvement	*GW2*, *GW5*, and *TGW6*	Improvement of grain weight	OsUbi OsU3, OsU6, TaU3	OsUbi OsU3, OsU6, TaU3	Agrobacterium-mediated transformation	[22]
	*Hd2*, *Hd4*, and *Hd5*	Early maturity of rice varieties	*Cas9* Pubi-H	OsU3/U6a	-	[118]
	*GS9*	Yield Improvement	CaMV 35S	OsU3	Agrobacterium-mediated transformation	[119]
	*OsGRF4*	Yield Improvement	2 × 35S	OsU6	Agrobacterium-mediated transformation	[120]
	*TMS5*	Photoperiod controlled male sterile lines	-	OsU3/U6	Agrobacterium-mediated transformation	[121]
	*TMS10*	Photo- and thermosensitive	-		Agrobacterium-mediated transformation	[122]
	*CSA*	Photoperiod-controlled male sterile lines	-			[123]
	*Gn1a*	Grain number; Panicles	ZmUbi	U6a	Agrobacterium-mediated transformation	[120]
	*DEP1*	Plant height; Erect panicles; Grain size				
	*GS3*	Grain size				
	*IPA1*	Plant height and tiller number				
	*CCD7* *PYLs*	Increased tiller number; Improved growth and productivity	OsUbi	*OsU3*	Agrobacterium-mediated transformation	[124]
	*Lazy1*	Pronounced tiller spreading	OsUbi OsU3	OsUbi OsU3	Agrobacterium-mediated transformation	[35]
	*OsPDS*, *OsBADH2*, *Oso2g23823*, *OsMPK2*	Tolerance capacity against various abiotic stress factors	2 × 35S	OsU6	Particle bombardment	[4]
	*ISA1*	Quality improvement	CaMV 35S	OsU6	Agrobacterium-mediated transformation	[125]
	*YSA*	Young albino seedlings	CaMV 35S	OsU6–2	Agrobacterium-mediated transformation	[126]
	*PDS*	Phytoene Desaturase				
	*DL*	Drooping Leaves				
	*Chlorophyll A oxygenase (CAO I)*	Pale green leaves	OsUbi	OsU3	Agrobacterium-mediated transformation	[35]
	*ROC*	Outermost Cells	CaMV 35S	OsU6–2	Agrobacterium-mediated transformation	[19]
	*OsCYP97A4*, *OsDSM2*, *OsCCD4a*, *OsCCD4b*, *OsCCD7*	Quality improvement	CaMV 35S	OsU3	Agrobacterium-mediated transformation	[126]
	*BADH2*	Enhanced fragrance	CaMV 35S	OsU3	Agrobacterium-mediated transformation	[127]
Biotic stress tolerance	*OsERF922*	Enhanced resistance to blast disease	-	-	Agrobacterium-mediated transformation	[20]
	*OsAnn3*	Tolerance to cold stress	CaMV 35S	OsU6	Agrobacterium-mediated transformation	[7]
	*Bsrk1*	Disease resistance	CaMV 35S	OsU6	Agrobacterium-mediated transformation	[128]
	*OsSWEET13*	Bacterial blight disease resistance	-	-	-	[129]
	*OsSWEET11*, *OsSWEET14*	Bacterial blight disease resistance	ZmUbi	OsU3	Agrobacterium-mediated transformation	[4,34]
	*Xa13*	Bacterial blight disease resistance	CaMV 35S	OsU3 & OsU6	Agrobacterium-mediated transformation	[130]
	*eIF4G*	Resistance to rice *tungro spherical* virus	ZmUbi	TaU6	Agrobacterium-mediated transformation	[131]
	*OsMPK5*	Various abiotic stress tolerance and disease	CaMV 35S	OsU6	Agrobacterium-mediated transformation	[36]
	*Acetolactate synthase (ALS)*, *DNA Ligase 4*	Disease resistance	2 × 35S	OsU6	Expression plasmid vectors	[10]
Abiotic stress tolerance	*BEL*	Herbicide-resistant	2 × 35S	AtU6–26	Agrobacterium-mediated transformation	[21]
	*OsNAC041*	Salinity	-	-		[9]
	*OsEPSPS*	Glyphosate-resistant	CaMV 35S	OsU3	Agrobacterium-mediated transformation	[132]
	*ALS*	Herbicide-resistant	2 × 35S	OsU6	Agrobacterium-mediated transformation	[133]
	*Bentazon Sensitive Lethal*	Phenotypic analysis showed plants susceptible to bentazon	-	-	Agrobacterium-mediated transformation	[21]
	*OsDERF1*, *OsPMS3*, *OsEPSPS*, *OsMSH1*, *OsMYB5*	Drought tolerance	CaMV 35S, OsUBQ1	OsU6 OsU3	Agrobacterium-mediated transformation	[134]
	*OsPYL*	Drought tolerance	-	-	-	[124]
	*OsAOX1a*, *OsAOX1b*, *OsAOX1c*	Various abiotic stress tolerance	2 × 35S	AtU6–26	Agrobacterium-mediated transformation	[135]
	*OsHAK-1*	Low cesium accumulation	CaMV 35S	OsU6a	Agrobacterium-mediated transformation	[136]
	*OsPRX2*	Potassium deficiency tolerance	-	-	-	[137]
	*OsSAPK2*	Drought tolerance	-	-	-	[6]
Nutritional improvement	*OsNramp5*	Low cadmium content	CaMV 35S	OsU6a	Agrobacterium-mediated transformation	[8]
	*ISA1*	Starch	CaMV 35S		Agrobacterium-mediated transformation	[125]
	*OsWaxy*	Amylose synthase	CaMV 35S	OsU6	Agrobacterium-mediated transformation	[138]
	*SBEIIb* and *SBEI*	Generation of high amylose rice	ZmUbi	OsU3	Agrobacterium-mediated transformation	[139]
	*lysC* and *dapA*	Lysine content	*CaMV 35S*	-	Agrobacterium-mediated transformation	[140]
Stomatal density	*OsEPFL9*	Regulates stomatal leaf density	-	-	-	[141]
Cyclin-dependent kinase	*CDKA1*, *CDKA2*, *CDKB1*	-	CaMV 35S	OsU3	Agrobacterium-mediated transformation	[142]
	*CDKB2*	-	2 × 35S	OsU3	Agrobacterium-mediated transformation	[143]
Homologous pairing activity	*OsDMC1A*, *OsDMC1B*	Disrupted meiotic cDNA	2 × 35S	OsU3	Agrobacterium-mediated transformation	[144]

‘–’: information unavailable.

## Data Availability

Not applicable.

## Abbreviations

2AP:2-Acetyl-1-Pyrroline; A: Adenine; ABE: Adenine Base Editors; APOBEC: apolipoprotein B mRNA editing enzyme, catalytic polypeptide-like; bp: Base Pair; C: Cytosine; Cd: Cadmium; CMV: Cauliflower Mosaic Virus; CRISPR/Cpf1: Clustered Regularly Interspaced Short Palindromic Repeat From Prevotella And Francisella 1; CRISPR: Clustered Regularly Interspaced Short Palindromic Repeat; Cas9: CRISPR Associated Endonuclease 9; crRNA: CRISPR Derived RNA; DSBS: Double-Stranded Break; G: Guanine; GET: Gene-Editing Technologies; HDR: Homology-Directed Repair; InDel: Insertion and or Deletion; N: Nucleotide; NHEJ: Non-Homologous End Joining; PAM: Protospacer Adjacent Motif; T: Thiamine; TALENs Transcription activator-like Effector Nuclease; TracrRNA: Trans-Activating CRISPR RNA; ZFNs: Zinc Finger Nuclease.
